# Early catheter ablation: A promising and effective approach for atrial fibrillation and post-heart failure recovery—Timing matters

**DOI:** 10.1016/j.ijcha.2025.101604

**Published:** 2025-01-09

**Authors:** Satoshi Yanagisawa, Yasuya Inden, Toyoaki Murohara

**Affiliations:** Department of Cardiology, Nagoya University Graduate School of Medicine, Nagoya, Japan; Department of Advanced Cardiovascular Therapeutics, Nagoya University Graduate School of Medicine, Nagoya, Japan; Department of Cardiology, Nagoya University Graduate School of Medicine, Nagoya, Japan; Department of Cardiology, Nagoya University Graduate School of Medicine, Nagoya, Japan

**Keywords:** Acute decompensated heart failure, Atrial fibrillation, Catheter ablation, Heart failure, Heart failure with preserved ejection fraction, Reverse remodeling

Atrial fibrillation (AF) and heart failure (HF) often coexist and exacerbate each other, resulting in a worsening prognosis and outcomes. According to large-scale cohort studies, AF was a complication in approximately 50 % of patients with HF, while a history of HF was present in about 20 % of patients with AF [1]. With longer life expectancies and improved survival rates, the global prevalence of these conditions is steadily increasing. In most cases, AF-associated HF manifests as heart failure with preserved ejection fraction (HFpEF), often without a clear history of structural heart disease. This presentation is more common than heart failure with reduced ejection fraction (HFrEF) [2].

The management of AF is increasingly shifting toward early rhythm control therapy, despite limited evidence from the past two decades. The Atrial Fibrillation Follow-Up Investigation of Rhythm Management (AFFIRM) trial previously found no significant difference in mortality between rhythm- and rate-control strategies [3]. However, recent evidence from the Early Treatment of Atrial Fibrillation for Stroke Prevention Trial (EAST-AFNET 4) demonstrated that early rhythm control therapy in patients with newly diagnosed AF and pre-existing cardiovascular comorbidities significantly improved outcomes, including reduced rates of cardiovascular death, stroke, or hospitalization for HF or acute coronary syndrome. These findings highlight the benefits of early rhythm control therapy, even though many strategies involve antiarrhythmic drugs [4].

In the EAST-AFNET 4 study, 38 % of patients experienced their first episode of AF, and 36 % had paroxysmal AF, with a median time from diagnosis to intervention of 36 days. This underscores the importance of prompt diagnosis and early rhythm management, regardless of whether the therapy is invasive or noninvasive. Advances in ablation technology, such as cryoballoon ablation, have shown significant benefits as a first-line therapy compared with antiarrhythmic drugs, particularly in reducing AF recurrence [5]. Early catheter ablation using these advanced technologies may offer a feasible and effective approach for patients with HF and AF, potentially improving outcomes and prognoses in terms of both efficacy and safety.

Several retrospective studies have explored the advantages of early ablation for patients with HF and AF. Tóth et al. reported a significant reduction in AF recurrence when ablation was performed within 12 months of diagnosis compared to after 12 months in 227 patients with left ventricular ejection fraction (LVEF) < 50 % [6]. Similarly, Ando et al. found that cryoballoon ablation within six months of diagnosis significantly reduced AF recurrence rates in a Japanese multicenter study involving 543 HF patients [7]. Subanalyses of the EAST-AFNET 4 trial also demonstrated substantial benefits of early rhythm control therapy in improving primary endpoints for patients with HF [8]. However, no randomized clinical trials have specifically evaluated early catheter ablation for AF in patients with HF, leaving the optimal timing for such interventions uncertain. Further research is needed to address this gap and provide clearer guidance for clinical practice.

In this issue of *IJC Heart & Vasculature*, Che et al. [9] retrospectively investigated the prognostic effects of early catheter ablation for persistent AF in patients with acute decompensated heart failure (ADHF) at a single center in China. The study analyzed 50 matched pairs of patients using propensity score matching, comparing those who underwent early ablation with those who underwent elective ablation. Early ablation was performed once ADHF symptoms stabilized prior to discharge, whereas elective ablation was scheduled for patients who were initially discharged after ADHF control and later readmitted for ablation following a transitional period of 90 days. The mean time from ADHF onset to ablation was 10.6 ± 2.5 days for the early group and 158.3 ± 84.5 days for the elective group. During the one-year follow-up, the early ablation group demonstrated a significantly lower rate of HF hospitalization. Notably, rehospitalization rates were significantly higher during the 90-day transitional period in the elective group compared to the early group. Subgroup analysis revealed that these benefits were more pronounced in patients with HFrEF compared to those with HFpEF. The findings of this study highlight the potential advantages of early AF ablation immediately following recovery from ADHF, offering new insights into early rhythm control in HF management. These results suggest an opportunity to broaden therapeutic strategies from an alternative perspective.

Heart failure is a progressive condition characterized by acute exacerbations and recurrent hospitalizations, which gradually impair cardiac function and increase stiffness, leading to poor outcomes and reduced physical activity. The interplay between AF and HF exacerbates this decline, creating a self-perpetuating cycle often summarized as “AF begets HF, and HF begets AF.” HF contributes to the development of AF through several mechanisms such as elevated left atrial pressure, which induces atrial fibrosis and scarring [10]. This results in reduced conduction velocity and prolonged action potential duration, particularly for decreased LVEF, all of which facilitate conduction block, reentry initiation, and sustained AF [11], [12]. Additional mechanisms, such as the activation of the renin-angiotensin-aldosterone system and increased sympathetic activity, further contribute to AF pathogenesis [10].

Following recovery from ADHF and discharge, patients enter a vulnerable phase lasting 3–6 months, during which the risk of HF rehospitalization and mortality is elevated [13]. Several factors contribute to this increased risk. Subclinical hemodynamic abnormalities, such as elevated venous pressure at discharge, may not manifest as overt symptoms but still pose risks. Residual neurohormonal and inflammatory activity, evidenced by elevated but non-normalized brain natriuretic peptide and C-reactive protein levels at discharge, also plays a role [13]. Additionally, many cardioprotective drugs, namely guideline-directed medical therapy (GDMT), require gradual dose titration after initial low-dose administration. These low initial doses may be insufficient to prevent HF exacerbation. For patients with AF and HFpEF, the prognostic benefits of most GDMTs on mortality remain inadequately supported, emphasizing the need for further clinical studies. Delayed initiation and underdosing of GDMT are also common, underscoring the need for special attention during the vulnerable phase and alternative strategies to reduce the risk of HF rehospitalization.

In this context, the early catheter ablation approach for AF prior to discharge from ADHF, as proposed in the current study, may represent a promising option for improving outcomes during the vulnerable phase. In this study, only 4 % of patients in the early ablation group experienced HF rehospitalization during the transition period, compared to 26 % in the elective ablation group who experienced HF exacerbation before scheduled ablation. Regarding safety concerns, HF exacerbation resulted in delayed discharge for 8 % of patients in both groups. A total complication rate of 8 % in each group may be considered acceptable, particularly given recent advancements in catheter ablation techniques and imaging approaches that enhance the safety and efficacy of AF ablation performed immediately after ADHF recovery. These findings are further supported by a recent large-scale Japanese study, which demonstrated that catheter ablation for AF within 90 days of HF admission was associated with improved cardiovascular outcomes and reduced HF-related mortality [14]. Together, these results present a positive perspective on early AF ablation as a strategy to optimize outcomes for patients recovering from ADHF.

One limitation of this study was that most patients had a prolonged history of AF, exceeding 2–3 years (mean AF duration: 47.5 months in the early group and 43.6 months in the elective group) prior to undergoing ablation, with some patients having undergone previous ablation procedures. It remains unclear whether this extended period of persistent AF directly contributes to acute HF exacerbation, or whether catheter ablation offers significant benefits in the chronic phase of the disease. However, only 4 % of the patients in this study had a history of cardiomyopathy, which suggests that rhythm control interventions could benefit patients with undiagnosed cardiomyopathy, offering potential recovery of cardiac function even in cases of long-standing persistent AF [15]. Thus, the results of the current study highlight the advantages of AF catheter ablation shortly after ADHF recovery rather than focusing on the timing of ablation following AF diagnosis. Nevertheless, these results can also be considered in the context of early ablation strategies initiated soon after the onset of AF. Early catheter ablation may provide greater benefits both for patients immediately after ADHF stabilization and for those in the early stages of AF. Efforts to minimize the duration of persistent AF and reduce waiting times for ablation could further decrease HF readmissions and improve post-ablation outcomes.

## CRediT authorship contribution statement

**Satoshi Yanagisawa:** Writing – review & editing, Writing – original draft, Validation, Conceptualization. **Yasuya Inden:** Supervision. **Toyoaki Murohara:** Supervision.

## Funding

None

## Declaration of competing interest

The authors declare the following financial interests/personal relationships which may be considered as potential competing interests: Satoshi Yanagisawa reports a relationship with Medtronic Japan Co Ltd that includes: employment. If there are other authors, they declare that they have no known competing financial interests or personal relationships that could have appeared to influence the work reported in this paper.
